# The Relationship between Circulating ANGPTL8/Betatrophin Concentrations and Adult Obesity: A Meta-Analysis

**DOI:** 10.1155/2019/5096860

**Published:** 2019-10-22

**Authors:** Jingjing Ye, Yu Qin, Dong Wang, Ling Yang, Guoyue Yuan

**Affiliations:** Department of Endocrinology, Affiliated Hospital of Jiangsu University, 438 Jiefang Road, Zhenjiang, Jiangsu 212001, China

## Abstract

In this study, we evaluated the relationship between circulating betatrophin levels and obesity. Obesity is a common public health problem that is increasing globally. Betatrophin, a newly identified protein, is predominantly expressed in white and brown fat tissues and in the liver. Growing evidence suggests that betatrophin plays a pivotal role in metabolism, including the synthesis and degradation of lipids in cells, and adipocyte differentiation. Previous studies have assessed the association between circulating betatrophin levels and obesity; however, this relationship remains unclear. Therefore, our study is aimed at examining the impact of betatrophin on obesity using a meta-analysis of the current evidence. We performed a meta-analysis to quantify the relationship between betatrophin levels and obesity. A literature search was conducted through the EMBASE, Web of Science, and MEDLINE databases. Retrieved studies were screened, without any language restrictions to identify relevant literature published up to December 2018. Observational studies, in which the association between circulating concentrations of betatrophin and obesity was evaluated, were considered suitable for the systematic review. Of the 65 manuscripts retrieved, 9 datasets from 6 studies, involving 681 participants, detected an association between circulating betatrophin and obesity. Circulating betatrophin levels of obese subjects were higher than those of nonobese subjects (random − effects weighted mean difference (WMD) = 0.250 *μ*g/mL, 95% CI: 0.048–0.451, *I*^2^ = 94.8%, *p* = 0.015), yet with significant between-study heterogeneity. This heterogeneity appeared to be modified by glycemic status but not by age, the ELISA kits used, sample source, or body mass index. The high circulating betatrophin concentration may directly increase the risk of obesity in adults. Betatrophin may serve as a therapeutic target for obesity in adults.

## 1. Introduction

Obesity causes considerable health and socioeconomic problems in many countries. Previous epidemiological studies have reported conflicting results regarding betatrophin levels and obesity, but the biological mechanisms underlying this relationship remain obscure.

Obesity is a common public health problem and is on the rise worldwide. This is apparent from the 2014 World Health Organization (WHO) report, which stated that over 0.6 billion people were obese, and more than 1.9 billion adults were overweight [1]. In recent years, obesity has become a significant burden on global health care expenditures. It is frequently associated with chronic disorders, including heart disease, polycystic ovary syndrome, breast cancer, and diabetes. Globally, obesity and overweight contribute to at least 2.8 million deaths each year [2].

Adipose tissue, recognized as an important endocrine organ, has attracted much attention due to its effect on few metabolic processes such as glucose homeostasis, lipid metabolism, inflammation, and blood pressure. Betatrophin, also known as “ANGPTL8,” “RIFL,” “C19ORF80,” “TD26,” and “lipasin,” is a novel adipokine secreted from the adipose tissue and the liver [3]. Recent studies have indicated that betatrophin is linked to glucolipid disorders [4]. In this case, circulating betatrophin levels might be affected by nutritional status that alters the body weight [5]. The study done by Ren et al. on mice demonstrated that the transcript level of angiopoietin-like protein 8 (ANGPTL8) was higher for subjects with obesity [6]. However, the findings of multiple epidemiological studies have not been entirely consistent. Several studies showed that circulating betatrophin levels were not significantly different in adults with obesity [7, 8]. By contrast, some studies demonstrated a positive relationship between circulating betatrophin levels and obesity [9–12], whereas other studies demonstrated an inverse relationship [13–15]. Hence, more reliable association between circulating betatrophin concentration and the risk of obesity is warranted.

Thus far, a meta-analysis has not been performed specifically to assess the association between circulating betatrophin concentration and the risk of obesity. Therefore, the aim of the present study was to systematically review all eligible studies that have assessed the association between betatrophin levels and the risk of obesity, thereby providing a quantitative estimation of the association.

## 2. Materials and Methods

### 2.1. Ethical Approval and Patient Consent

This study is a meta-analysis. Hence, approval by an ethics committee and informed patient consent were not required.

### 2.2. Data Sources

A comprehensive literature search was performed in the EMBASE, Web of Science, and MEDLINE databases up to December 2018, without any language restrictions. The search strategy included the following query terms: “ANGPTL8,” “betatrophin,” “RIFL,” “C19ORF80,” “TD26,” “lipasin,” “obesity,” and “obese.” Furthermore, the reference lists of the identified papers were reviewed for comprehensive search results. The study was conducted in accordance with the MOOSE group standards for reporting meta-analysis of observational studies. Titles, abstracts, and full-text papers were screened by 2 independent reviewers with expertise in conducting systematic reviews. Disagreements were resolved by consulting a 3rd reviewer, who was not involved in the initial procedure.

### 2.3. Study Selection

#### 2.3.1. Inclusion and Exclusion Criteria

Observational studies were considered suitable for the systematic review, in which the association between circulating concentrations of betatrophin and obesity was evaluated. If conference abstracts included sufficient data to extract effect estimates, they were also included. The exclusion criteria were as follows: (1) review paper, (2) search not restricted to humans, and (3) studies lacking a nonobese control group. For overlapping publications, the most complete data were included.

#### 2.3.2. Data Extraction

Data from each eligible study were independently collected by two researchers, using a predesigned data extraction form. Any disagreements were checked by a third investigator. General characteristics of each study were extracted: year of publication, the first author's last name, the mean age and body mass index (BMI), country, cases and control or cohort size, glycemic status, and betatrophin measurement method. The quality of each study was evaluated using elements of the Newcastle-Ottawa Scale (NOS) [16]. In addition, when data were missing or when there was doubt regarding information in the publications, we communicated with the authors to obtain further information.

#### 2.3.3. Statistical Analysis

The data on betatrophin levels in each study were adopted to mean difference ± standard deviation (SD). Standard error of the mean was converted to SD. The weighted mean differences (WMDs) of total betatrophin levels were calculated for all the eligible studies in the meta-analysis and were used in fixed-effects or random-effects models. The *χ*^2^ test and *I*^2^ test evaluated heterogeneity in the results of the studies (a *p* < 0.1 and *I*^2^ > 50% was considered to indicate a statistically significant heterogeneity) [17]. Visual inspection of publication bias by a funnel plot of the data was not applied, as it may be interpreted incorrectly if studies were heterogeneous [18, 19]. Begg's funnel plot and Egger's regression asymmetry test was used to estimate the publication bias [20]. Moreover, sensitivity analysis was undertaken with the exclusion of the studies with borderline eligibility. Subgroup analyses were performed by geographic region, sample source, the studies employing different betatrophin ELISA kits, and the studies with different mean age, BMI, and glycemic status. The statistical analysis and meta-analysis were performed with Stata 11.0 (Stata, College Station, TX, USA). The present study was conducted in accordance with the MOOSE guidelines for meta-analysis [21].

## 3. Results

### 3.1. Search Results

**Figure 1:**
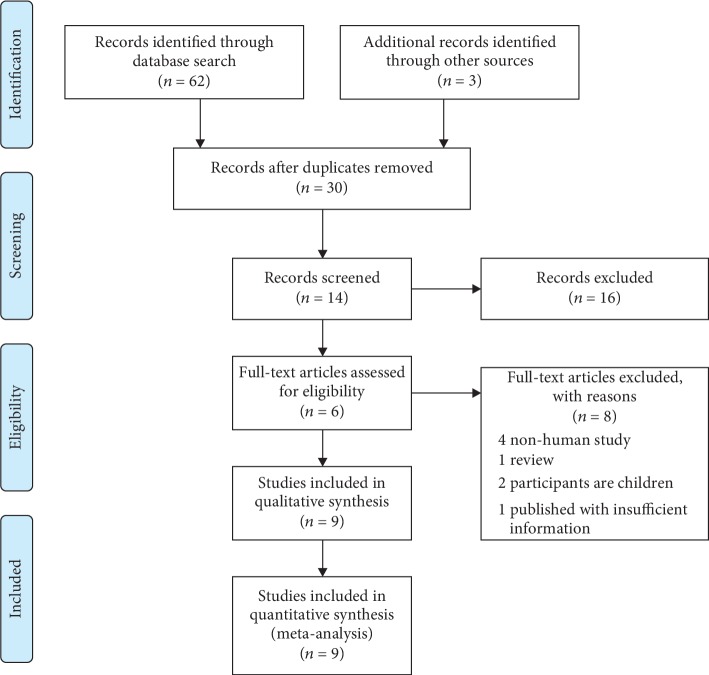
PRISMA flow diagram of the literature search and selection process.

A PRISMA flow chart of the search procedure is presented in Figure 1. The search strategy identified 65 potentially relevant papers, with 59 being excluded after deduplication and title/abstract screening. After further analysis of the identified papers, we finally included 9 datasets from 6 studies (2 papers reported results for 5 separate studies) [7, 8, 10–12, 14].  Table 1 presents the main characteristics of the 9 studies. Only circulating full-length betatrophin concentration was measured in these studies.

### 3.2. Main Analysis

**Figure 2:**
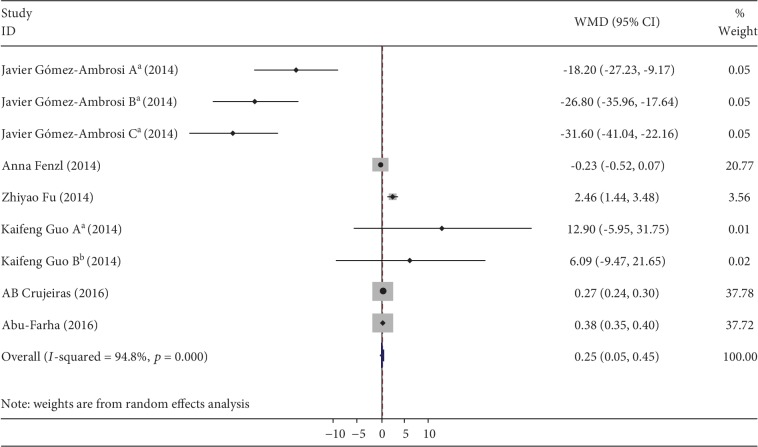
Forest plot for the association of circulating betatrophin levels and obesity. The summary estimates were analyzed using a random-effects model. (a) The study by Gómez-Ambrosi et al. [14], where A represents data from individuals with normal glycemic values, B from individuals with impaired glucose tolerance, and C from individuals with diabetes mellitus. (b) The study by Guo et al. [8], where A represents data from individuals with normal glycemic values and B from individuals with diabetes mellitus.

The meta-analysis included 9 datasets from 6 studies [7, 8, 10–12, 14]. Overall, 307 healthy controls and 374 patients with obesity were included in the meta-analysis. Cochran's *Q* test was used to assess the heterogeneity among the 9 studies. Since significant heterogeneity was found between the studies (*Q* = 154.39, *d*.*f*. = 8, *p* < 0.05), the random-effects model was adopted. All of the studies were included in the random-effects model. We observed that circulating betatrophin levels of obese subjects were higher than that of nonobese subjects (random-effects WMD = 0.25 pg/mL, 95% CI: 0.048–0.451, *I*^2^ = 94.8%, *p* = 0.015).  Figure 2 shows the WMD and 95% CIs for each individual study.

Since there was a high level of heterogeneity among the included studies, we subsequently performed subgroup analyses by all characteristics to assess whether they can explore the potential source of heterogeneity between circulating betatrophin levels and obesity. Subgroup analyses indicated that potential variables such as age, different sample sources (serum vs. plasma), different betatrophin ELISA kits (ELISA kits from Wuhan EIAAB Science vs. other companies), and geographical area demonstrated no significant difference (Table 2). However, when we stratified the results by glycemic status, this indicated that glycemic status may have partially been the source of heterogeneity.

We further conducted a sensitivity analysis to assess the stability of these results. The sensitivity analysis was conducted by sequentially deleting each study to check the influence of an individual study on the results. The conclusions of the sensitivity analysis revealed no significant alteration in the direction of effect, which indicated the stability of our meta-analysis.

Begg's test and Egger's regression asymmetry test were conducted to evaluate the publication bias. Begg's test (*p* = 0.452) and Egger's regression asymmetry test (*p* = 0.297) demonstrated that publication bias was not detected.

## 4. Discussion

Betatrophin, a newly founded adipocytokine, was believed to play a pivotal part in metabolism. Betatrophin was a major focus in obesity research, for which relevant research results have been published continuously. Several epidemiologic studies have demonstrated that there is an independent and positive association of circulating betatrophin with obesity, whereas other studies reported no association. Inconsistent results were obtained because of the relatively small sample size in the studies, which may be affected by numerous factors. Hence, we conducted a meta-analysis to further evaluate the relationship between circulating betatrophin levels and obesity.

The present study is the first comprehensive meta-analysis that quantitatively explores a possible relationship between circulating betatrophin levels and obesity. Our study showed that circulating betatrophin levels increased in individuals with obesity.

Obesity has increased rapidly over the past few decades, which is associated with a significantly increased risk of premature death. The increasing prevalence of obesity is a challenge to public health care in both developed and developing countries. This obesity epidemic is a major driving force for the increase in the risk of chronic metabolic complications, including type 2 diabetes [22]. Recent studies have demonstrated that a number of adipokines (adiponectin, vaspin, leptin, chemerin, etc.) have been associated with inflammatory responses and metabolic disorders in patients with obesity [23]. However, few data are available regarding betatrophin.

Betatrophin, also called as angiopoietin-like protein 8 (e.g., ANGPTL8), hepatocellular carcinoma-associated protein TD26 and chromosome 19 open reading frame 80 (C19orf80), TD26, refeeding induced fat and liver (RIFL) protein, and lipasin, is a novel protein predominantly secreted in the liver and the adipose tissue. Previous studies in mouse models implicated an important role for betatrophin in a number of metabolic-related pathways, such as lipid metabolism and energy balance in mice [10]. In this meta-analysis of 9 observational studies, individuals with obesity had higher betatrophin levels. There is emerging evidence that supports a positive relationship between circulating betatrophin and obesity. In vitro studies have shown that betatrophin expression increased over 100-fold during 3T3-L1 cell adipogenesis. Its knockout in 3T3-L1 cells during adipogenesis and its knockdown leads to a reduction in adipogenesis [6]. Furthermore, betatrophin expression was increased by approximately 8 times in white adipose tissue (WAT) of ob/ob mice (an obesity mouse model), when compared with wild-type mice [24]. With these striking studies, many scholars conducted research examining the relevance between betatrophin and obesity by detecting circulating betatrophin levels in human subjects. Similarly, betatrophin mRNA is highly expressed in humans with obesity [25]. Mounting evidence has indicated that high circulating betatrophin levels represent a predisposing status for the development of obesity. The observed positive relationship is likely to be mediated by 3 possible biological mechanisms. First, betatrophin levels have a close relationship with inflammatory markers, including high-sensitivity C-reactive protein (HsCRP) levels [26], which is central to the development of obesity [27]. Second, betatrophin overexpression was shown to relate to hypertriglyceridemia, while the absence of betatrophin was shown to decrease triglyceride levels without affecting glucose levels [28, 29]. Third, insulin resistance and enhanced insulin contribute to the upregulation of betatrophin levels [30, 31]. Inflammation, lipid metabolism, and insulin resistance are potential modifiable risk factors for obesity [32–34]. Given the connection between obesity and inflammation, lipid metabolism, and insulin resistance, it is obvious that high circulating betatrophin levels are positively correlated with obesity risk.

Since a substantial heterogeneity existed among the analyzed studies, we analyzed the potential contributing factors that might explain why the results varied between the studies. Subgroup and sensitivity analyses were conducted to investigate the potential sources of heterogeneity. BMI is frequently used to measure obesity but does not seem to offer an explanation for this heterogeneity. Betatrophin is predominantly secreted by the liver in humans. Some studies have shown that serum betatrophin levels were significantly higher in patients with nonalcoholic fatty liver disease (NAFLD) compared to those without NAFLD. However, BMI failed to account for the accumulation of fat within the liver. A previous study showed that a higher percentage of visceral fat typically correlates with a higher risk of NAFLD [35]. More studies are needed to elucidate the relationship between circulating betatrophin levels and visceral fat.

Recently, several researchers have raised a concern regarding the potential influence of glycemic status differences on betatrophin levels [36, 37]. Stratified subgroup analyses were conducted to evaluate between-study variations in glycemic status. Notably, the impact of glycemic status seems to partially explain the heterogeneity observed. However, the underlying mechanisms remain unknown and need to be elucidated. Sample source and the ELISA kits used are considered to affect measured betatrophin levels [9, 38]. However, our findings showed that neither were contributing factors to the heterogeneity between studies. In addition, we used a sensitivity analysis in a stepwise manner to reanalyze the statistics of each paper. The results show that the meta-analysis results are stable and provide more reliable interpretation results.

There are several limitations in the present study, which should be addressed in accounting for these results. First, there was a lot of variation in sample sizes of the included studies, which might impact the statistical heterogeneity observed in our meta-analysis. Therefore, high-quality studies consisting of sufficient sample sizes were needed for further evaluation regarding the association between circulating betatrophin levels and adult obesity in the future. Second, the study is based on observational studies, which are particularly vulnerable to information or selection bias. Third, exclusion of grey literature may increase the risk of publication bias. However, no sign of such bias was detected in either Begg's test or Egger's regression asymmetry test. Fourth, different forms of betatrophin display different roles, but in our study, only full-length betatrophin levels were detected in the study population. Fifth, circulating betatrophin concentration might be affected by sex or lipid profiles, which cannot be taken into account because of lack of data. Finally, only fasting, circulating betatrophin was detected. As a food intake-induced hormone, postprandial betatrophin after a standard diet could be more meaningful to be analyzed in future investigations [39].

## 5. Conclusions

In summary, this meta-analysis demonstrated that circulating betatrophin levels in patients with obesity are higher than those of healthy controls. Thorough understanding of the role of betatrophin in the progression of obesity is helpful to better guide patient treatment. Moreover, further studies are required to explore the possible physiologic mechanisms of betatrophin and its role in obesity.

## Figures and Tables

**Table 1:** Characteristics of studies included in the meta-analysis.

Study	Country	Sample	Case group				Control group		
			Glycemic status	*n*	Betatrophin (ng/mL)	Mean BMI (kg/m^2^)	*n*	Betatrophin (ng/mL)	Mean BMI (kg/m^2^)
Gómez-Ambrosi et al. (2014 A) [14]^a^	Spain	Serum	NG	75	26.9 ± 15.4	39.4 ± 6.7	33	45.1 ± 24.4	23.6 ± 1.2
Gómez-Ambrosi et al. (2014 B) [14]^a^	Spain	Serum	IGT	30	18.3 ± 10.7	43.7 ± 7.5	33	45.1 ± 24.4	23.6 ± 1.2
Gómez-Ambrosi et al. (2014 C) [14]^a^	Spain	Serum	T2DM	15	13.5 ± 8.8	39.0 ± 3.6	33	45.1 ± 24.4	23.6 ± 1.2
Fenzl et al. (2014)	Austria	Plasma	NG	19	0.973 ± 0.402	46.9 ± 1.4	20	1.2 ± 0.54	24.6 ± 0.6
Fu et al. (2014)	USA	Serum	NG	29	4.42 ± 0.58	—	24	1.96 ± 0.98	—
Guo et al. (2015 A) [8]^b^	China	Serum	NG	30	33.91 ± 46.64	32.95 ± 2.84	17	21.01 ± 18.25	22.35 ± 1.48
Guo et al. (2015 B) [8]^b^	China	Serum	T2DM	19	27.1 ± 28.59	33.47 ± 2.96	17	21.01 ± 18.25	22.35 ± 1.48
Abu-Farha et al. (2016)	Kuwait	Plasma	NG	62	1.15 ± 0.108	34.76 ± 3.22	82	0.775 ± 0.046	24.66 ± 2.86
Crujeiras AB et al. (2016)	Spain	Plasma	Unknown	95	1.24 ± 0.43	35.7 ± 4.5	48	0.97 ± 0.69	22.9 ± 2.2

BMI: body mass index; NG: norm glycemic; IGT: impaired glucose tolerance. Data are presented as means ± SD. ^a^From the same study. ^b^From the same study.

**Table 2:** Meta-analysis of circulating betatrophin levels and obesity by study characteristic.

Characteristic	No. of studies	Case	Control	WMD	(95% CI)	Heterogeneity tests
Geographic region						*I* ^2^
Europe	4	215	147	-18.76	(-37.049, -0.465)	0.96
Asia	3	111	116	1.16	(-2.579, 4.891)	0.096
Other	2	48	44	1.11	(-1.522, 3.744)	0.99
Sample source						
Serum	6	198	157	-9.972	(-25.131, 5.187)	0.955
Plasma	3	176	150	0.172	(-0.123, 0.467)	0.878
Mean BMI (kg/m^2^)						
<35	3	111	116	1.16	(-2.579, 4.891)	0.096
≥35	5	234	167	-2.408	(-4.114, -0.703)	0.96
Unknown	1	29	24	2.460	(2.015, 2.905)	0.00
ELISA kit						
Wuhan EIAAB Science Co.	3	110	126	0.541	(-0.077, 1.158)	0.946
Other	6	264	181	-10.478	(-24.627, 3.67)	0.948
Mean age						
≥45	6	230	222	-7.656	(-11.645, -3.667)	0.955
<45	2	49	37	-0.224	(-0.521, 0.074)	0.465
Unknown	1	95	48	0.270	(0.244, 0.296)	0.0
Glycemic status						
NG	5	215	176	0.534	(-0.313, 1.380)	0.92
Hyperglycemia	3	64	83	-18.547	(-37.148, 0.054)	0.88
Unknown	1	95	48	0.270	(0.244, 0.296)	0.00
